# Field evaluation of near point of care Cepheid GeneXpert HIV-1 Qual for early infant diagnosis

**DOI:** 10.1371/journal.pone.0209778

**Published:** 2018-12-27

**Authors:** Valarie Sarah Opollo, Alliance Nikuze, Jihane Ben-Farhat, Emily Anyango, Felix Humwa, Boaz Oyaro, Stephen Wanjala, Willis Omwoyo, Maxwel Majiwa, Victor Akelo, Clement Zeh, David Maman

**Affiliations:** 1 Kenya Medical Research Institute/Centre for Global Health Research, Kisumu, Kenya; 2 Epicentre, Paris, France; 3 Médecins Sans Frontières, Nairobi, Kenya; 4 Ministry of Health, Homabay, Kenya; 5 U.S. Centers for Disease Control and Prevention, Clinical Research Center, Kisumu, Kenya; Waseda University, JAPAN

## Abstract

**Background:**

Access to point-of-care HIV testing shortens turn-around times, time to diagnosis and reduces loss to follow-up hence minimizing barriers to early linkage to care and treatment among HIV infected infants. Currently samples for early infant HIV diagnosis are sent to centralized testing facilities which are few and located only at specific regions in Kenya. However, there are Point of Care (POC) early infant diagnosis [EID] technologies elsewhere such as SAMBA and ALERE-Q that are yet to be evaluated in Kenya despite the urgent need for data to inform policy formulation regarding EID. The Cepheid GeneXpert HIV-1 Qual (GeneXpert) technology for POC EID offers a great opportunity to minimize HIV associated morbidity, mortality and loss to follow-up through decentralization of early infant HIV testing to the clinics. This technology also allows for same-day results thus facilitating prompt linkage to care.

**Methods:**

We evaluated the GeneXpert HIV Qual EID POC in Homabay County against the standard of care platform, Roche CAP/CTM HIV-1 qualitative PCR, using dried blood spots (DBS). Between February—July 2016, DBS samples were collected from HIV exposed children <18 months of age enrolled in a cross-sectional study. Samples were collected by qualified nurse counselors, and were tested by trained technicians using field based GeneXpert and conventional laboratory based Roche CAP/CTM HIV-1 qualitative PCR. Sensitivity and specificity were determined.

**Results:**

Overall, 3,814 mother/infant pairs were included in the study, out of which 921 infants were HIV exposed as per the mothers’ HIV status and based on the infant’s HIV rapid test. A total of 969 PCR tests were performed, out of which 30 (3.3%) infants were concordantly positive using both platforms. GeneXpert HIV-1 Qual yielded a sensitivity of 94.1% and specificity of 99.8% with an overall error rate of 0.7%.

**Conclusion:**

Our findings show that GeneXpert HIV-1 Qual performs well compared to CAP/CTM using DBS samples, suggesting that this technology may be adopted in decentralized laboratories as a near POC device. It may contribute to prompt diagnosis of HIV exposed infants hence enabling early linkage to care, thus advancing further gains in EID.

## Introduction

In 2016, 1.8 million children were living with HIV globally, with 98,200 living in Kenya [1], majority were infected by their mothers during pregnancy, childbirth or breastfeeding [2]. The expansion of the prevention of mother-to-child transmission (PMTCT) programs, has seen a decrease of new infections among children by 50% since 2010, with a further decline by 2015, with 150,000 children newly infected with HIV globally [3]. However in Kenya, about 5% MTCT still occur [1]. Perinatal HIV infection contributes to an increase in morbidity and mortality among children in their first years of life [4], and lack of antiretroviral treatment (ART) and late ART initiation has seen more than half of the infants infected with HIV die before their second birthday [5–10]. The first step in the provision of early infant diagnosis [EID] services and linkage to care among HIV exposed infants is early identification; however this still remains a challenge in sub-Saharan Africa [11]. In an effort to reduce childhood mortality, the world health organization [12] recommends early detection of HIV among infants within six weeks of life and prompt ART initiation to all children under ten years. [13]. Access to HIV diagnosis for HIV-exposed infants and children is a challenge in SSA because of the processes involved in HIV detection that require use of nucleic acid amplification tests conducted by trained personnel in centralized laboratories usually based in urban centres [14, 15]. Other multiple factors also contribute to delay in diagnosis among HIV–exposed infants and these include lack of decentralized sample collection sites, stock outs of HIV supplies, poor sample transfer and referral networks and long turn-around times [TATs] from sample collection to return of results to clinicians. Several policy and program initiatives through the global public health community advocating for an increase in treatment access to HIV infected children exist. These include U.S. President’s Emergency Plan for AIDS Relief (PEPFAR) partnership with the Children’s Investment Fund Foundation (CIFF) for the Accelerating Children’s HIV/AIDS Treatment [5] Initiative and World Health Organization [12] guidelines that recommend for universal ART eligibility for all children. These initiatives focus on identifying HIV infected children and ensuring that early HIV diagnosis is conducted among those who are exposed. Despite these initiatives, access to EID is only available to very few children, who actually need the test in resource limited settings (RLS), with only 42% children who are HIV-exposed having received a virologic test within the first two months of life in 2014 [16]. Adoption of dried blood spots as sample of choice for HIV diagnosis among infants was a major step in expanding early HIV testing [17]. Nevertheless, major operational challenges still exist around sample preparation, the long TATs and poor retention of infants who access care [14, 15, 18, 19]. Majority of available testing options are laboratory based technologies that require sophisticated equipment, dedicated space and well trained users [20]. Additionally, centralized testing is infrastructurally expensive, often with long TATs because of the capital investments, extensive specimen and result transport networks involved [14, 20, 21]. This has proven to be a challenge in RLS since they limit access to EID and contribute to loss to follow-up among those children who are able to access care.

An alternative to these conventional platforms include the point of care (POC) or near POC devices that are simple, reliable and of high quality and have a potential of expanding EID to low level health facilities through decentralization. POC technologies can be used as a tool to monitor the quality of prophylaxis [22] and would contribute to improved TATs and addressing gaps in sample collection and transfer. Additionally, POC devices may lead to improved access to testing and linkage to care hence allowing for same day or faster clinical decision making [23]. This would subsequently improve retention into care [24], through improved ART scale-up and decentralized testing contributing to the attainment of UNAIDS 90-90-90 targets by 2020. Moreover, using simplified technologies in HIV diagnosis especially in field settings has the potential to evade a number of logistical challenges faced by laboratory-based EID systems [25], and this will potentially contribute to reduced diagnostic and monitoring costs for people living with HIV/AIDS [22]. Recent evidence on POC EID technologies have focused on laboratory-based evaluations, where conditions are ideal for testing and may not expose the testing gaps in clinical settings within the region, few POC devices that have been evaluated in field-settings are not clear on acceptability of the EID POC devices [24, 26]. The GeneXpert HIV-1 Qual Assay was prequalified by WHO in 2016 for EID. It is an easy to use, near POC device (i.e., intended for use in laboratories where electricity is accessible, cannot be operated at primary healthcare settings with no electricity) and can be operated by personnel with minimum laboratory training and produces results within a short TAT. It offers an ideal opportunity and solution for scaling up EID in RLS since it is a compact device that requires minimum training to operate and provides accurate and timely results. Uptake of near POC technologies require clearly mapped evaluations that prove their suitability and use by focusing on its performance characteristics such as sensitivity and specificity compared against conventional standards such as the centralized gold standard technologies. The GeneXpert HIV-1 Qual Assay with GeneXpert POC machine is CE marked but not yet validated in Kenya. We evaluated the performance of the GeneXpert HIV-1 Qual system in a field setting and compared the results with the current EID conventional standard in western Kenya.

## Methods

### Setting

Ndhiwa is one of the eight sub-counties of Homabay County in western Kenya and has an overall population of 172,212 [27]. It is located in the Nyanza region, the area of Kenya most affected by HIV, with an estimated prevalence of 15.1% in 2011 [28, 29]. Ndhiwa was among the first places in Kenya to introduce PMTCT option B+ in 2014. At the time of the study, Ndhiwa sub-county had 33 health facilities offering ART and PMTCT services but only 26 public facilities were selected for participation; including one sub-county hospital, 3 health centers and 22 dispensaries. Within the 26 health facilities, the study created 4 hubs–the hubs were central health facilities/laboratories which acted as near POC testing points for EID. Ndhiwa was implementing the local guidelines of PMTCT option B+ involving universal initiation of ART for all HIV-infected pregnant women [30]. The Impact of Expanded Screening Strategies (IESS) study was a facility-based survey with a prospective follow-up of HIV+ infants. Mothers seeking immunization services for their infants aged between 2 to 10 weeks and 8 to 10 months were enrolled between 1st February and 28th July 2016. Infants aged 0 to 18 months coming for consultation, out-patient department (OPD) and in-patient department (IPD) services, as well as mothers delivering in the maternity of Ndhiwa hospital were also eligible.

### Design

We performed a field evaluation of GeneXpert HIV-1 Qual (Cepheid, Sunnyvale, CA, USA) comparing the POC technology with the gold standard for EID PCR, COBAS AmpliPrep/COBAS TaqMan HIV-1 Qualitative Test, v2.0 assay (CAP/CTM HIV-1) qualitative PCR (Roche diagnostics, Branchburg, NJ, USA). The study was conducted among mother/guardian-infant pairs attending expanded programs of immunization (EPI) services at selected clinics and maternity at Ndhiwa sub-county hospital. Eligible infants attending EPI were those aged 6 weeks (+/- 4weeks) and 9 months (+/- 1month) and all infants born in the maternity’s hospital. Mother-baby pairs were excluded mainly because of the age of infants, did not consent or were disabled. Samples were collected from HIV-exposed children attending the health facilities at all these service points. The study assumed a mean MTCT rate of 8% during the study period with an expected HIV-prevalence among children of 1.76%.

### Study procedures

The child was tested regardless of the mother/guardian status in order to compare HIV exposure defined by mother’s reported status and child’s positivity to HIV rapid test, as recommended in the WHO guidelines [31]. Routinely, DBS is collected for PCR testing through finger or heel prick and spotted directly onto the filter paper, however in this study whole blood EDTA sample (≤1ml) was collected by finger stick or heel prick at the health facilities because the same sample was used for screening the infants and spotting two filter papers, a procedure that is not routinely done at the health facilities. 75μl of the blood was spotted onto Whatman 903 pre-printed circles following routine programmatic testing, an additional DBS was prepared using the same blood draw and couriered the same day to the hubs that were used as testing laboratory using the near POC. Rapid HIV testing was done using serial tests with Determine Rapid HIV-1/2 Antibody test (Abbott Rapid Diagnostics) followed if positive by Unigold Rapid HIV test kits (Trinity Biotech PLC). The filter paper were air-dried at the health facilities and transported daily to laboratory hubs where the POC GeneXpert devices were placed and for temporary storage in preparation for transport to the KEMRI HIV research laboratory in Kisumu where routine EID was conducted. The DBS were transported at room temperature and tested within 72hours of collection. Briefly, the GeneXpert technology is a closed, self-contained, fully-integrated and automated platform that is designed to purify, concentrate, detect and identify targeted nucleic acid sequences using nested real-time reverse transcriptase polymerase chain reaction (PCR) assays, thereby delivering results directly from unprocessed samples. The system has a modular design. Patient samples are added to disease-specific, single use, disposable cartridges, containing all PCR reagents, buffers, primers and probes, and these are then loaded into one of the system’s modules. The test cartridge also contains an internal control to verify adequate processing of the target sequence and the system monitors the presence of inhibitors in the PCR assay to avoid a false-negative result. Test results (detected or not detected), as well as assay details including sample adequacy are accessed from a computer connected to the system. Results can be stored and viewed at the convenience of staff at any time after the test is completed. Trained health care workers or lab technicians are required to operate the device. Both CAP/CTM HIV-1 and GeneXpert HIV-1Qual assays were conducted according to manufacturer’s recommendations. During the study period, the GeneXpert HIV-1 Qual Assay was performed on six different GeneXpert instruments by 10 trained laboratory technologists. The field-based laboratory technologists had formal training provided by the manufacturer on use of the POC, where they participated in a three day training program by the manufacturers before the field evaluation began. For sample analysis, one spot of the DBS was placed into the sample reagent buffer tube and incubated in the thermomixer for 15 min at 56°C then rotated at 500rpm; 1ml of the liquid was transferred to the cartridge and closed, finally the cartridge was loaded in the POC instrument and result read. The test took approximately 90 minutes to process. The result was considered valid when the cartridge built-in controls were passed by the instrument. When there was a quality control failure due to sample or instrument errors, an error code was provided and the data was recorded by the laboratory technologist and a repeat testing of sample with POC assay error was performed. All study samples were tested on both the Standard of Care (SOC) and POC platforms, and staff running both platforms were blinded to the test results. We repeated any sample associated with a discordant SOC/POC result, some of them necessitated further tests with additional samples. During analysis, these repeated tests were classified as second runs whereas the initial runs were classified as first runs. POC results were not given back to the study participants since the test was not approved locally and therefore was considered as experimental, therefore POC testing did not influence the clinical management of the participants and health care providers offered routine care as per usual practice. For confidentiality purposes, the specimens were de-identified before testing could take place.

### Analysis

All POC results were entered into a Microsoft excel database (Microsoft Corporation, Redmond, WA, USA). Data was analysed using STATA version 13 for Windows. Participant characteristics were presented by use of frequencies and percentages for categorical data, median and interquartile range for continuous data. Sensitivity and Specificity (with 95% Confidence intervals) of GeneXpert HIV QUAL EID POC were compared to Roche CAP/CTM HIV-1 qualitative PCR which was taken as the reference testing method.

### Assessing operational characteristics

The laboratory technologists using the POC in the field were assessed on their feedback on using the new technology, focusing on the operational characteristics, ease of use and technique of the POC. This information was collected using a self-administered questionnaire and the information was recorded manually and analyzed descriptively.

### Ethical approval

The study was approved by the Kenya Medical Research Institute Ethical Review Committee (reference KEMRI/RES/7/3/1—Non SSC Protocol No.507) and the Comitteé de Protection des Personnes d’Ile de France (reference 15077). Written informed consent was obtained when mothers/guardians presented at the study service points for routine immunization and at the maternity department.

## Results

### Characteristics of study population

**Fig 1:**
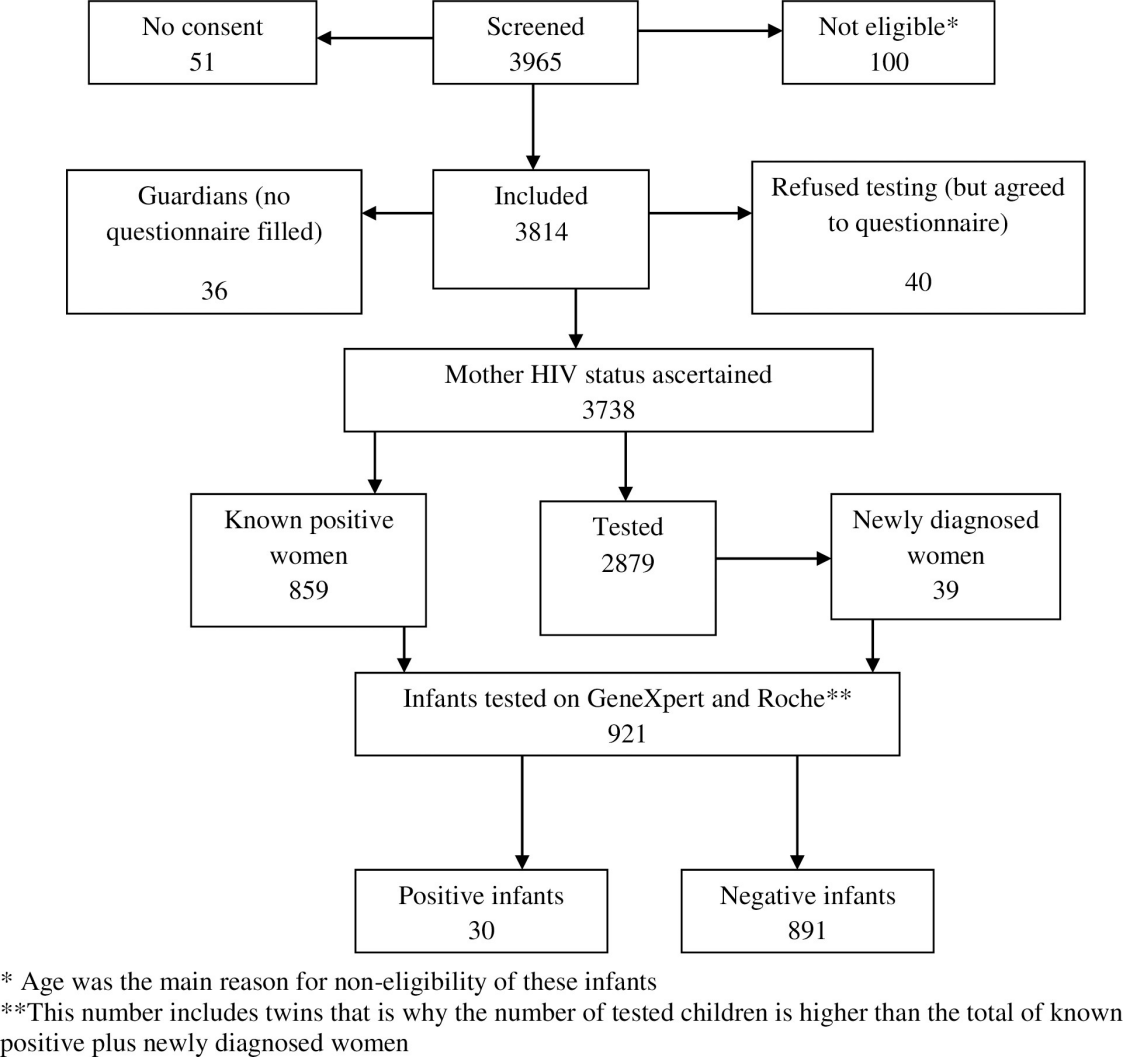
Inclusion flow chart It shows the characteristics of the study population, including children and women who were part of the study.

A total of 3965 mothers were screened, of whom, 3814 (96.2%) were included in the study. Of them, 3,738 mothers had their HIV status ascertained, as 40 mothers refused testing and 36 infants were not with the mother but with a guardian (usually the grandmother). Of them, 859 were already known positive and 2,879 women were thus tested for HIV– 39 were newly diagnosed (Fig 1). Using HIV status of the 898 mothers to define HIV-exposure, 921 (24.1%) of children were exposed to HIV while using the Infant HIV Determine test results to define exposure, 559 (14.3%) babies were categorized as exposed (Table 1). A total of 969 POC tests were performed on 921 [24.6% (95% CI 23.2–26.0)] children who were HIV exposed. Approximately 15% of the children were tested as newborns and 49% of them tested at routine EID visit of 6 weeks. Out of the 921 exposed infants identified throughout the study, 30 were found HIV positive through both the Roche CAP/CTM and near point of care (GeneXpert), leading to a MTCT rate of 3.3% (95% CI 2.3–4.7).

**Table 1:** Summary of HIV exposed children by service.

	Included Mothers (n)	Prevalence N (%)	Included Infants (N)	HIV exposure by mother self-report status (N/%)	HIV exposure by Infant Determine test (N/%)	Positive N (MTCT rate %)
Total	3814	898 (23.5)	3919	921 (24%)	559 (14.3%)	30 (3.3)
**Service**						
EPI 6 weeks	1879	409 (21.9)	1920	420 (22.4)	344 (17.9)	14 (3.3)
EPI 9 months	1019	300 (30.1)	1056	306 (30)	89 (8.4)	10 (3.3)
OPD	300	45 (15.5)	311	49 (16.3)	12 (4.0)	4 (8.2)
Maternity	536	126 (23.6)	545	128 (23.9)	110 (20.2)	2 (1.6)
IPD	80	18 (23.7)	87	18 (22.5)	4 (4.8)	0 (0.0)

### Sensitivity and specificity of POC test

**Fig 2:**
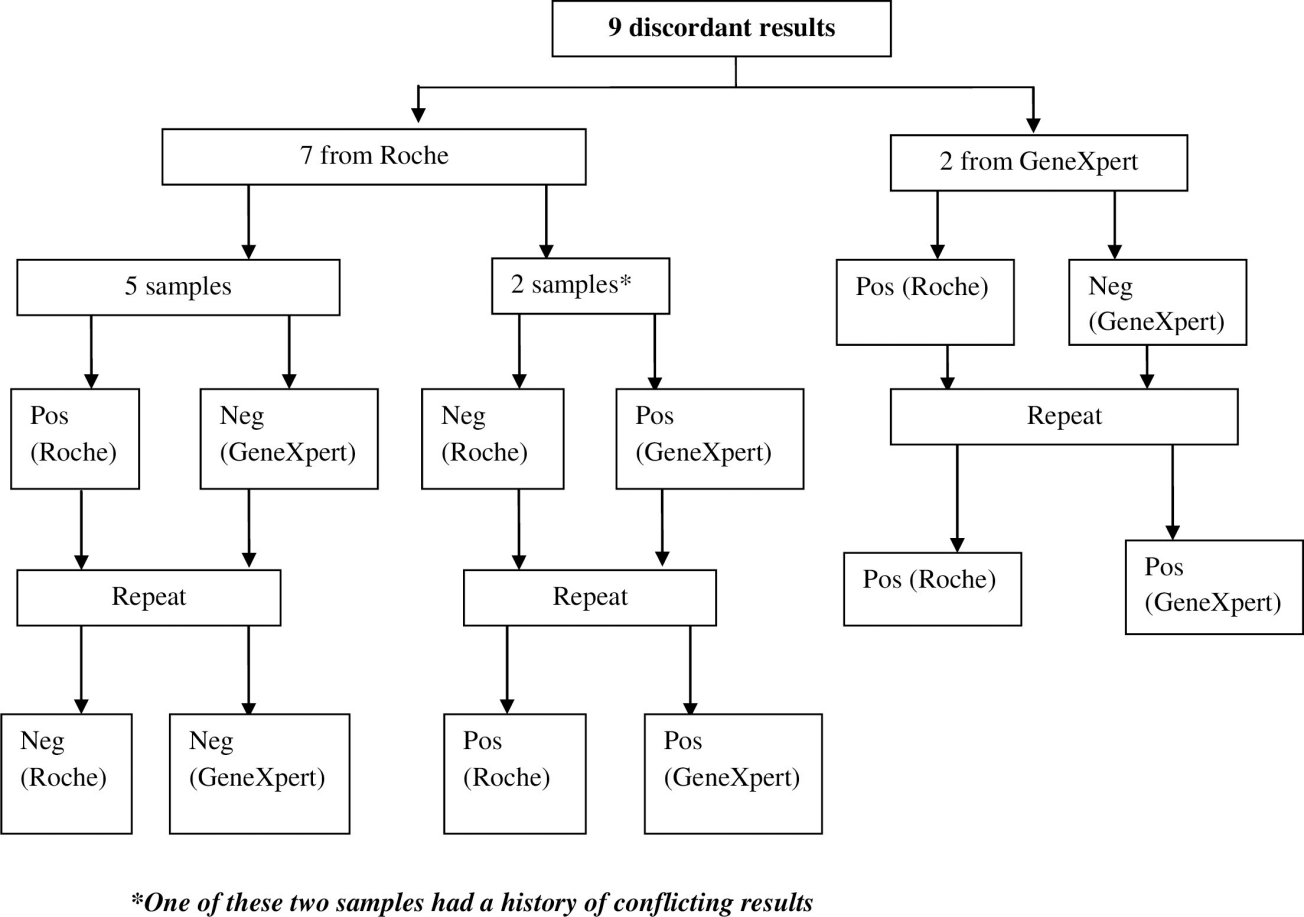
Discordant results It shows the 9 discordant cases observed in the study, the resolutions made to address them and the final outcome.

Results of the POC sensitivity and specificity compared with the SOC are shown in Table 2. A total of 969 results were correctly assayed, with a final sensitivity and specificity of 94.1% and 99.8% (95% CI) respectively, with two false negative PCR found (Table 2). The POC gave an error rate of 0.7%, (7/969 samples)–all were resolved after a repeat test. On analysis of the first test results, 960/969 (99%) POC results were concordant with the SOC results. The specificity of the POC was 99% and a sensitivity of 82%, thus drawing attention to 9 discordant cases that were retested on both platforms on a second run; out of those 9 cases, 7 were from Roche platform and 2 were from the GeneXpert (Fig 2).

**Table 2:** Performance of Cepheid GeneXpert HIV-1 Qual (first and second runs) against Roche CAP/CTM HIV-1 PCR.

	First run	Second (Final) run
Number of tests	969	969
Number of HIV +ve tests	39	34
% of tests HIV +ve	4.0%	3.3%
Sensitivity	82.1%	94.1%
Specificity	99.8%	99.8%
Positive predictive value	94.1%	94.1%
Negative predictive value	99.3%	99.8%
Machine error rate	0.7%	

Five samples were first found negative on GeneXpert but positive on Roche. The discordance was on the Roche platform. When a new sample was collected, the tests were negative on Roche (and still negative on GeneXpert). If Roche had worked correctly, we would have had a sensitivity of 94.1% as opposed to the 82% that we found on the first runs.

Two samples were negative on Roche but positive on GeneXpert but a repeat on the same blood on Roche gave a positive result. The discordance was on the Roche platform. Had it been in the routine program, Roche, the standard of care, would have missed two positive infants! However, one of these two samples had a history of conflicting results: positive on Roche and negative on GeneXpert on the 1st and 2nd blood collected but on the 3rd blood collected, it was positive on GeneXpert and negative on Roche first but then a repeat on Roche also turned positive. It is possible that the 1st and 2nd blood was mislabeled and/or contaminated and that the transmission from mother to infant happened thereafter.

Two samples were negative on GeneXpert and positive on Roche. On GeneXpert, those two samples had their results reading as “HIV-1 NOT DETECTED” on GeneXpert, the samples were in fact positive on a repeat test of the same sample for one and positive on, the second blood collected for the other (first sample/DBS card was exhausted while repeating the test due to numerous power interruption). In the plotted result graph, the HIV primary curve started to show albeit late. While reading a result, the laboratory technician or any other provider, should read both the graph and test result to determine the positivity of a sample.

After repeat running to resolve assay discrepancies and inconclusive SOC results through additional testing, results were available for the 921 HIV-exposed children.

### Operational characteristics

Staff using the POC device stated that only one DBS spot or 100ul whole blood was required to perform the EID test on the device. The hands on time for starting up the device and running controls for the POC ranged between 25 to 30 minutes, with 4 samples being tested per every run, and the total time required to run the test was 90 minutes to obtaining test result. Average sample throughput was 8 sample runs per day with all hubs receiving at least 15 samples per day. The device was easy to use and instructions for both device and test were easy to follow and interpret, with further provision of pausing the device without affecting the test in case there was need. Once the test was completed the result was displayed on the computer screen ready for printing. There were no breakdowns over 969 module runs experienced for the POC although there was back-up equipment available in case there was need for it. The study staff operating the POC identified a few advantages of the device, including the use of DBS, as a sample type with limited sample volume, to run the test, limited specimen preparation for DBS, the device was hands-on with limited user engagement, and the ability to test at least 4 samples simultaneously on the different modules with detailed results printout and stored electronically within the POC device. However they also noted a few difficulties in interpreting the graph for the EID test result, a few cases of increased temperatures within the POC devices, a longer run time per test and frequent power fluctuations although batteries were available as power back-up. Overally the staff reported that the GeneXpert was a high performing device that was easy and feasible to use within the field setting.

## Discussion

These results evaluate the performance of GeneXpert POC HIV-1 Qual by comparing its results to the current standard of care platform for EID and after repeat testing and confirms that GeneXpert POC technology can be successfully used outside in the field environment to provide accurate values for infants who are HIV exposed. This is the first field evaluation of the GeneXpert POC device in this region to diagnose HIV among children. HIV detection in children during their first years of life can be life-saving especially when the children are put on ART, however the current diagnostic systems are slow due to centralized testing and hence delayed prophylaxis among the children with frequent loss-to-follow up as well as missing results for more than 50% of children tested [32].

It is important to diagnose HIV-exposed children as soon as possible and ensure that they are initiated on treatment fast [22]. Our findings show that GeneXpert had overall high concordance with the SOC with very high sensitivity and specificity after repeat testing of all discordants in the field setting and only one pending discordant result, supporting previous studies on the GeneXpert HIV-1 Qual [33–35] assays. This is important because the effectiveness of POC technologies in field settings depends on their implementation and burden load to the existing health system infrastructure [36]. This is further highlighted by increased task shifting and decentralization of testing from central laboratories to field-based laboratories.

High specificity rates are critical in the use of technologies that are used in diagnosing infants who are HIV exposed. The specificity of the POC test remained high and steady at 99%, with two false positive results detected and this uncertainty was resolved after follow-up testing; this highlights the importance of the validation process since the discordance between the two platforms allowed for further testing on the same sample with eventual concordant results being found; it is possible the false positive results may be related with the process of sample preparation in the hubs prior to loading in the cartridge. These findings are consistent with the manufacturers’ report of GeneXpert performance [37]. It is important to conclusively close-out such discordances since maternal and child health through PMTCT has positively evolved, with a decrease in incidence witnessed, and especially in the study setting (where HIV prevalence among children was estimated to be 1.76%) where this decrease has contributed to an increasing concern among false positive PCR results and hence ensuring that EID technologies that maximizes on ruling out specificity are available is an important advancement in MTCT as noted by this POC device.

The sensitivity of the POC was stable compared to the SOC platform, indicating that the POC was equally able to capture all the true positives, and this was dependent on the sample volume used to run the assay. The sample input of 75ul for DBS is convenient for EID use, and is similar to the SOC sample requirement in sample volume. However, the one case of false negative sample observed on the POC assay hints that the sample generated a low CT value which could have impacted on the SOC positive result that was missed by the POC assay. Detection of low copy numbers of HIV is a major challenge facing EID assays, particularly during ART prophylaxis for children.

As explained in detail in the results section above, the study found a number of discordant results that had to be retested as out of 9 discordant results with Roche, 7 were Roche (considered as “gold standard”) errors, however reasons for this discordance need to be investigated further and addressed. It is key that technicians using GeneXpert HIV-Qual do not only read the results but also look at the graphs, which will be difficult in real conditions.

The study also witnessed a few machine errors during testing, which was leaning on a lower side, 0.7% (n = 10) compared to errors reported in previous POC field evaluation studies [26, 35]. All errors were resolved through retesting. However the errors may be attributed to issues experienced in studies conducted earlier and was resolved before the initiation of this study; the same can be said about POCT training that may have improved since earlier studies, addressing the common errors and how to evade them [38]. Therefore this does not raise a major alarm as plans for implementing the GeneXpert are being underway; most were attributed to sample quality during specimen collection and user errors. However such failures and errors could impact on feasibility and costing of delivering EID assays through POC technologies and hence future evaluations should focus on a step by step guide on addressing and mitigating such errors.

There were a few strengths observed in the study, however, the greatest strength of study was that it was conducted in a field setting where there are plans to implement and scale-up the POC devices, this allowed for this POC EID technology to be evaluated using fresh samples in a real world setting as opposed to centralized laboratories which are modernized. The study was also conducted by laboratory staff that had limited hands-on and formal training on PCR, however the experiences in performing the POC assays offers a positive indication of feasibility and ease of use of these devices once implementation and scale-up occurs. This analysis focused mostly on DBS as a sample which is most preferred for EID, and since the sensitivity of whole blood is higher than DBS the POC was able to capture all the positives and the negatives that were captured by the SOC assay. The study had some limitations as well. Although the GeneXpert was operated as a POC device, it is in fact a near POC technology since it was placed and operated from the hubs. This therefore means that in as much as the POC technology contributed to increased access to EID and testing, and generation of faster results, the issue of TAT needs to be looked at keenly since it is one of the benefits of a POC technology. However, we have observed from the findings that the GeneXpert can be successfully used in a field setting, and if used as a near POC, it will greatly improve on service delivery and access to testing in a region that is in dire need of more technologies to aid in HIV diagnosis among infants. It was not possible to retest all the concordant results between the two platforms as a quality assurance measure and therefore any shared false positive/negative results could have been missed.

In conclusion, this study has demonstrated that the GeneXpert POC EID assay has a high sensitivity and specificity and performs well in a field setting with high coverage of ART prophylaxis among mothers and children. The sensitivity and specificity of 94% and 99% respectively, does reflect the true performance of the device even though we had 9 discordant results with Roche, 7 were Roche (considered as “reference test”) errors. Nevertheless, it is key that technicians using GeneXpert HIV-Qual do not only read the results but also look at the graphs, which will be difficult in real conditions. This study has shown that POC testing complements centralized laboratory testing, is important for field situations and may solve the problem faced by centralized laboratories in meeting the TAT; especially in situations that require rapid TAT of results for clinical decisions.

Finally, although the evaluation period was short the POC users reported ease of POC use and were confident that the GeneXpert assay is appropriate for field settings and could be scaled-up in the health facilities for routine EID testing. These findings are line with WHO ‘ASSURED ‘criteria on POC devices that require them to be affordable, sensitive, specific, user-friendly, robust, equipment-free and deliverable to those who need it [12].
